# A predictive signal model for dynamic cardiac magnetic resonance imaging

**DOI:** 10.1038/s41598-023-37475-5

**Published:** 2023-06-25

**Authors:** Aaron D. Curtis, Alexander J. Mertens, Hai-Ling Margaret Cheng

**Affiliations:** 1 The Edward S. Rogers Sr. Department of Electrical and Computer Engineering, University of Toronto, Toronto, Canada; 2 Translational Biology & Engineering Program, Ted Rogers Centre for Heart Research, Toronto, Canada; 3 Institute of Biomedical Engineering, University of Toronto, 661 University Avenue, Room 1443, Toronto, Ontario M5G 1M1 Canada

**Keywords:** Electrical and electronic engineering, Magnetic resonance imaging, Biomedical engineering

## Abstract

Robust dynamic cardiac magnetic resonance imaging (MRI) has been a long-standing endeavor—as real-time imaging can provide information on the temporal signatures of disease we currently cannot assess—with the past decade seeing remarkable advances in acceleration using compressed sensing (CS) and artificial intelligence (AI). However, substantial limitations to real-time imaging remain and reconstruction quality is not always guaranteed. To improve reconstruction fidelity in dynamic cardiac MRI, we propose a novel predictive signal model that uses a priori statistics to adaptively predict temporal cardiac dynamics. By using a small training set obtained from the same patient, the new signal model can achieve robust dynamic cardiac MRI in the presence of irregular cardiac rhythm. Evaluation on simulated irregular cardiac dynamics and prospectively undersampled clinical cardiac MRI data demonstrate improved reconstruction quality for two reconstruction frameworks: Kalman filter and CS. The predictive model also works with different undersampling patterns (cartesian, radial, spiral) and can serve as a versatile foundation for robust dynamic cardiac MRI.

## Introduction

Cardiac MRI (CMR) is an important clinical tool for assessing cardiac 3D anatomy, mechanics, and tissue microstructure and function^1,2^. Unfortunately, challenges such as long acquisition times and the ability to capture irregular cardiac dynamics still remain. Because only a small fraction of the information necessary for a full resolution reconstruction can be acquired as the heart is beating, CMR currently gathers information pertaining to the same phase of the heart over multiple heart beats. This “averaging” over multiple cardiac cycles achieves high spatial resolution but also implies that scan times are long and true real-time dynamics are lost. This discarded information can be diagnostically relevant, especially when the dynamics are abnormal. For these reasons, there has been a long-standing effort to attain true real-time CMR^3–5^.

Multiple potent strategies have been attempted for real-time CMR^6^. These strategies are *k-t* acceleration, compressed sensing (CS), and artificial intelligence (AI). With *k-t* acceleration techniques, linear minimum mean-squared error (LMMSE) estimation is utilized to facilitate image reconstruction and guarantee reconstruction quality. As a recent example, the Kalman filter, known for real-time estimation and tracking of signals and objects^7,8^, has been applied to the dynamic CMR problem^9–11^. CS and AI technologies have also flourished, with CS seeing clinical implementation^12–14^. Unfortunately, each technique has inherent limitations. With *k-t* acceleration, irregular cardiac dynamics cannot be modeled and global structural details are neglected. In contrast, both CS and AI capture global details, but they may neglect local details^6^. Furthermore, in CS reconstruction, the undersampling rate is constrained to ensure fidelity^15,16^. In AI, wrong structures can be reconstructed if the acquired data falls outside the learned manifold^17^. Altogether, current acceleration strategies are susceptible to different sources of error.

We present a novel predictive signal model capable of adapting to the irregular cardiac dynamics often seen in real-world imaging of cardiac patients. The model is premised on the idea that reconstruction can be performed using statistical a priori information of what the heart *should* be doing at any given time. Thus, cardiac dynamics are not confined by an expectation of regular behavior. Our model was tested using both Kalman filtering and a state-of-the-art CS scheme based on GRASP^18^. We demonstrate that our predictive signal model improves image reconstruction quality in more realistic scenarios that mimic irregular cardiac dynamics. Lastly, we show that our approach is amenable to multiple k-space acquisition strategies. Our predictive signal model algorithms have broad implications by virtue of their real-time adaptation to unpredictable and changing cardiac dynamics, their versatile undersampling scheme, and their ability to be integrated with previously developed techniques for accelerated CMR.

## Theory

We will begin by deriving the predictive signal model within the context of the Kalman filter. We will then demonstrate how the predictive signal model may be used to augment a CS scheme.

### Kalman filter implementation

#### The Kalman filter for dynamic CMR

A Kalman filter consists of two key steps: a prediction step that uses a priori knowledge of signal dynamics, and a comparison between this prediction and observed data. In dynamic CMR, Kalman filters are derived from the following state-space equations:

1 $${x}_{t}={x}_{t-1}+{w}_{t-1},$$

2 $${z}_{t}=H{x}_{t}+{v}_{t},$$

where ${x}_{t}$ is the fully sampled (but unknown) CMR image at time $t$, ${w}_{t-1}$ is process noise, ${z}_{t}$ is k*-*space data acquired at $t$, $H$ is a measurement matrix relating ${x}_{t}$ to ${z}_{t}$, and ${v}_{t}$ is measurement noise. Equation (1) describes a priori knowledge of cardiac dynamics and assumes marginal dynamic changes by assuming the form of a random-walk process. Henceforth, we refer to previous work as a “random-walk” Kalman filter. Equation (2) describes the relationship between the fully sampled image and acquired k-space data and is required for the comparison step.

Acceptable performance of the random-walk Kalman filter demands robust estimation of the process noise covariance $Q$. However, obtaining an estimate in a realistic scenario may be challenging, especially if training data is limited. Furthermore, Eq. (1) assumes periodic cardiac motion^9,11^ and does not adequately model arrhythmia. A new format for Eq. (1) is necessary to achieve robust dynamic CMR. This is not a new concept and has been reflected in additional applications of Kalman filtering to MRI^19,20^.

#### Reworking the Kalman filter state-space model

We propose the following state-space model for Kalman filtering in dynamic CMR:

3 $${x}_{t}={f(x}_{t-1})+{w}_{t-1},$$

4 $${z}_{t}=EF{x}_{t}+{v}_{t},$$

where ${f(x}_{t-1})$ is a nonlinear transform of the previous state ${x}_{t-1}$ to account for non-marginal changes in cardiac dynamics. The measurement matrix $H$ has been replaced with $EF$, where $E$ is a downsampling matrix and $F$ is the 2D discrete Fourier transform. For non-cartesian trajectories, $EF$ would be equivalent to a non-uniform fast Fourier transform (NUFFT). In essence, acquired *k-*space (${z}_{t}$) will be interpolated onto a cartesian grid prior to the Fourier transform operation. Equations (3) and (4) yield the following Kalman filter equation^21^:

5 $${x}_{t}^{f}=f\left({x}_{t-1}^{a}\right),$$

6 $${P}_{t}^{f}={J}_{f}\left({x}_{t-1}^{a}\right){P}_{t-1}^{a}{J}_{f}^{H}\left({x}_{t-1}^{a}\right)+Q,$$

7 $$K_{t} = P_{t}^{f} F^{H} E^{H} (EFP_{t}^{f} F^{H} E^{H} + R)^{\dag } ,$$

8 $${x}_{t}^{a}={x}_{t}^{f}+{K}_{t}\left({z}_{t}-EF{x}_{t}^{f}\right),$$

9 $${P}_{t}^{a}=\left(I-{K}_{t}EF\right){P}_{t}^{f},$$

where ${x}_{t}^{f}$ is the predicted estimate of the fully sampled image ${x}_{t}$, ${J}_{f}({x}_{t-1}^{a})$ is the Jacobian of ${f(x}_{t-1})$ evaluated at ${x}_{t-1}^{a}$, ${P}_{t}^{f}$ is the predicted spatiotemporal covariance of ${x}_{t}$, superscript $H$ denotes the conjugate transpose, ${K}_{t}$ is the Kalman gain, $R$ is the covariance of the measurement noise, $\dag$ denotes the Moore–Penrose pseudoinverse, ${x}_{t}^{a}$ is the updated/actual estimate at time $t$, and ${P}_{t}^{a}$ is the updated/actual spatiotemporal covariance of ${x}_{t}$. Note the covariance of ${x}_{t}$ is spatiotemporal, as we are reconstructing a dynamic time-series.

**Figure 1:**
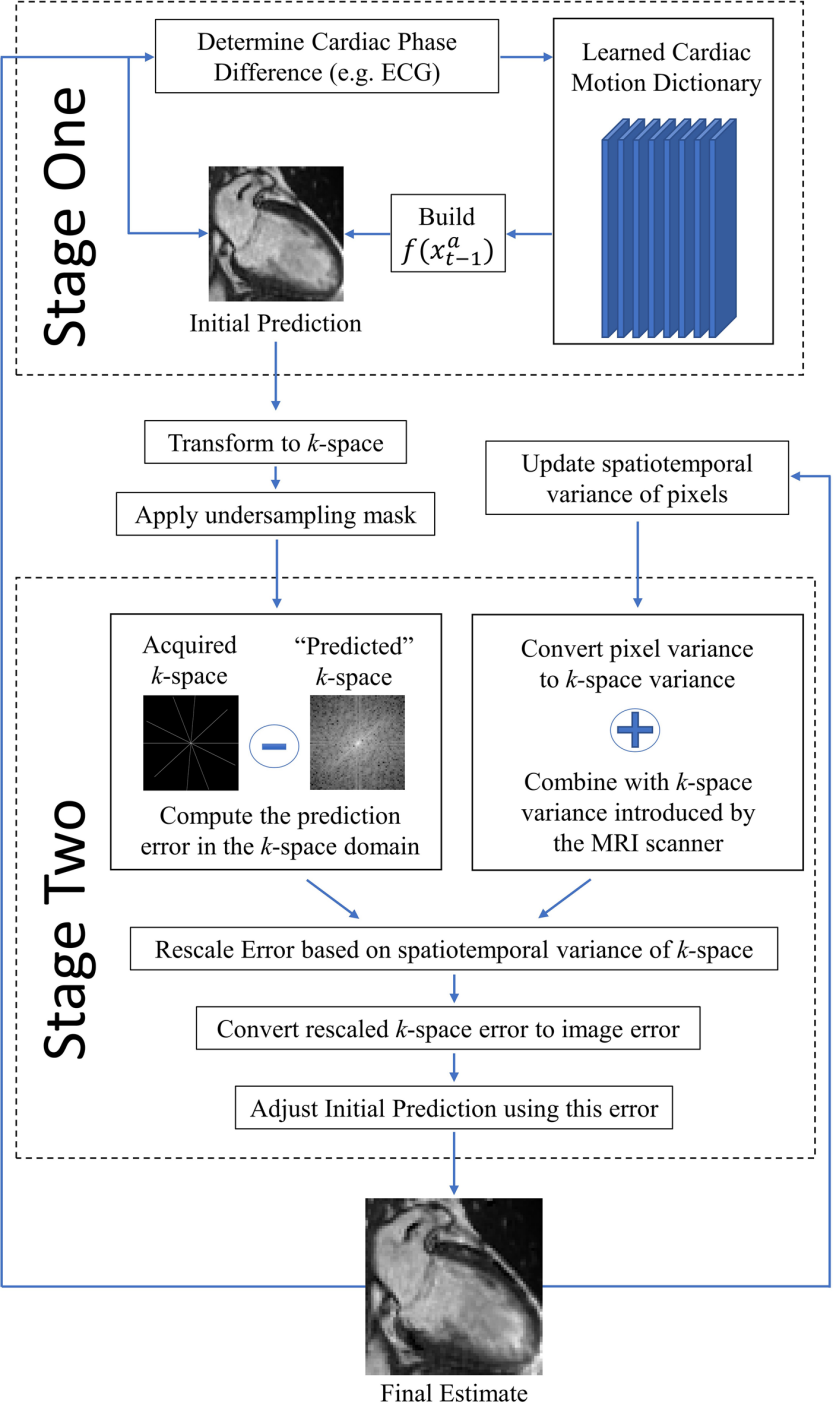
A predictive signal model incorporated in a two-stage Kalman filter. Stage One uses a learned cardiac motion dictionary to predict the next cardiac image. Stage Two uses the spatiotemporal statistics and the acquired *k-*space data to update this prediction and produce the final estimate.

To account for irregular cardiac behavior, we developed a statistical protocol for estimating $f({x}_{t-1}^{a})$ (Fig. 1). To mimic realistic scenarios, estimation was performed with limited training data consisting of a single fully sampled cardiac cycle. No additional cardiac cycles are required to estimate $f({x}_{t-1}^{a})$. We also assume the current cardiac phase is known (in practice, the cardiac phase can be determined via an electrocardiogram (ECG)). We will refer to Kalman filtering with a predictive signal model as a “two-stage Kalman filter”, where stage one is estimating $f({x}_{t-1}^{a})$ and stage two is the Kalman filter.

#### The predictive signal model: statistical estimation of ${f}({{x}}_{{t}-1}^{{a}})$

Consider a single arbitrary phase transition from ${x}_{t-1}$ to ${x}_{t}$. Suppose we wish to estimate ${x}_{t}$ via a linear combination of all pixels from the previous fully sampled image, ${x}_{t-1}$. As each pixel in ${x}_{t}$ has a non-zero mean, we will use an affine estimator:

10 $${x}_{t}={f}_{t}{x}_{t-1}+{b}_{t},$$

where ${f}_{t}$ is a state transition matrix for time $t$, and ${b}_{t}$ is a bias vector accounting for the non-zero means of ${x}_{t}$. Let us estimate ${f}_{t}$ and ${b}_{t}$ using temporal statistics, as estimating ${f}_{t}$ and ${b}_{t}$ using spatial statistics would require multiple images for each time point $t$. We will assume each pixel in ${x}_{t}$ is a temporal wide-sense stationary (WSS) random process. Thus, an $N\times N$ image consists of ${N}^{2}$ WSS random processes. Let us also assume these processes are jointly WSS. Using a previously acquired training scan consisting of a *single cardiac cycle* with $T$ fully sampled images (i.e. phases) of size $N\times N$, we can estimate the temporal covariance of each pixel and the temporal cross-covariance between each pair of pixels:

11a $${c}_{i,j}\left(l\right)=\frac{1}{T}\sum_{t=0}^{T-1}(B\left(i,j,t+l\right)-{\mu }_{i,j})(B(i,j,t{)-{\mu }_{i,j})}^{*},$$

11b $${c}_{(i,j)(p,q)}\left(l\right)=\frac{1}{T}\sum_{t=0}^{T-1}(B\left(i,j,t+l\right)-{\mu }_{i,j})(B(p,q,t{)-{\mu }_{p,q})}^{*},$$

where $l$ denotes the time delay, $B(i,j,t)$ denotes the value of pixel $(i,j)$ at time $t$, ${\mu }_{i,j}$ denotes the mean value of pixel $(i,j)$, and * denotes the complex conjugate. The temporal covariance and cross-covariance values are reorganized into $T$ matrices, where each matrix is indexed according to $l$:

12 $${C}_{l}=\left[\begin{array}{ccc}\begin{array}{cc}{c}_{\mathrm{1,1}}\left(l\right)& {c}_{\left(\mathrm{1,1}\right),\left(\mathrm{1,2}\right)}\left(l\right)\\ {c}_{\left(\mathrm{1,2}\right),\left(\mathrm{1,1}\right)}\left(l\right)& {c}_{\mathrm{1,2}}\left(l\right)\end{array}& \begin{array}{c}\cdots \\ \cdots \end{array}& \begin{array}{c}{c}_{\left(\mathrm{1,1}\right),\left(N,N\right)}\left(l\right)\\ {c}_{\left(\mathrm{1,2}\right),\left(N,N\right)}\left(l\right)\end{array}\\ \begin{array}{cc}\vdots & \vdots \end{array}& \ddots & \vdots \\ \begin{array}{cc}{c}_{\left(N,N\right),\left(\mathrm{1,1}\right)}\left(l\right)& {c}_{\left(N,N\right),\left(\mathrm{1,2}\right)}\left(l\right)\end{array}& \cdots & {c}_{\left(N,N\right)}(l)\end{array}\right].$$

Reorganizing the mean values into a single vector yields:

13 $${\mu }_{{x}_{t}}=\left[\begin{array}{c}{\mu }_{\mathrm{1,1}}\\ {\mu }_{\mathrm{1,2}}\\ \begin{array}{c}\vdots \\ {\mu }_{N,N}\end{array}\end{array}\right].$$

Notice that $l$ represents the *phase difference* between successive cardiac images. Thus, ${C}_{3}$ could describe a transition from cardiac phase 2 to 5. Assuming the current cardiac phase at time $t$ is known, the LMMSE estimator for ${f}_{t}$ and ${b}_{t}$ is as follows:

14a $${f}_{t}={C}_{l}({C}_{0}{)}^{-1},$$

14b $${b}_{t}=-{f}_{t}{\mu }_{{x}_{t}}+{\mu }_{{x}_{t}},$$

where $l$ is chosen based on the phase difference between $t-1$ and $t$. This LMMSE estimator is comprised of ${N}^{2}$ non-zero mean Wiener filters, one for each pixel in ${x}_{t}$. Refer to^22^ or k-t BLAST^23^ for more information regarding Wiener filtering. Equations (14a) and (14b) represent a learned cardiac motion dictionary. This dictionary is used to model the state transition between any two arbitrary cardiac phases. To understand how this works, recall Eq. (5): $f({x}_{t-1}^{a})$ is a non-linear transformation from ${\mathbb{C}}^{N\times N}$ to ${\mathbb{C}}^{N\times N}$. As the subspace does not change in this transformation, $f({x}_{t-1}^{a})$ can be represented as an affine transform. Thus, in our two-stage Kalman filter, we will set $f\left({x}_{t-1}^{a}\right)={f}_{t}{x}_{t-1}^{a}+{b}_{t}$. This implies ${J}_{f}({x}_{t-1}^{a})={f}_{t}={C}_{l}({C}_{0}{)}^{-1}$. We can now predict the next fully sampled CMR image using our dictionary, provided the phase difference is known. This prediction is then updated (Eqs. 7–9) using the spatiotemporal statistics of ${x}_{t}$. It is important to note that this prediction is an *approximation*: it need not perfectly represent the actual cardiac dynamics being imaged. To ensure our two-stage Kalman filter generalizes well, we developed additional protocols (see below) to enforce versatility.

#### Reconstructing multiple cardiac cycles

The dictionary was built assuming *WSS* and *jointly WSS* temporal statistics. As such, all autocorrelation and cross-correlation functions are conjugate symmetric:

15 $${C}_{-l}={C}_{l}^{H},$$

where ${C}_{l}^{H}$ denotes the conjugate transpose of ${C}_{l}$. The $-l$ corresponds to a negative time delay. We first consider a scenario where the training and test set both consist of *T* phases and we wish to reconstruct phase 1 of the next cardiac cycle using phase *T* of the current cycle. We can step *T* – 1 phases *backwards* in time by assigning ${C}_{l}={C}_{-(T-1)}$. Permitting backward transitions is what enables us to use a single cardiac cycle as our training scan. We can also apply backward transitions to arrhythmia: if the cardiac cycle abruptly returns to early systole (phase 1) from, say, early diastole (e.g. phase 7 out of *T*), we assign ${C}_{l}={C}_{-6}$ to move backwards 6 phases.

In any reconstruction, we must select $l$ based on which phase in the training scan bears the greatest resemblance to the currently estimated cardiac phase. This can be done by using ECG monitoring or non-ECG methods capable of tracking the sinus rhythm^24^.

#### Statistical estimation of Q

One caveat of using a single cardiac cycle in our training scan is that cardiac phases will look identical across multiple heartbeats (i.e. redundancy). An additional parameter is required to ensure our predictive signal model generalizes well: the process covariance, $Q$, where a non-zero value indicates the prediction from our dictionary only serves as an initial approximation.

It is critically important that $Q$ is defined judiciously. In previous work, $Q$ represents the covariance between two successive images in a periodic training scan^9,11^. In our work, we remove this assumption of periodicity by defining $Q$ as:

16 $$Q={{I}_{{N}^{2}\times {N}^{2}}{\circledast}C}_{0},$$

where ${C}_{0}$ is diagonalized to protect against overfitting. Note: ${C}_{0}$ may be computed directly from the training scan. Thus, only a single cardiac cycle is required to compute $Q$. Equation (16) mathematically admits that our dictionary may be incorrect, thereby conferring adaptability to our two-stage Kalman filter. Although $Q$ may have large diagonals (i.e. large power) that could result in image artifacts, $Q$ is naturally balanced by our dictionary where redundant terms produce an averaging/smoothing effect. Mathematically, our dictionary is an ${N}^{2}-1$ order finite impulse response (FIR) lowpass filter.

#### Accommodating for low temporal resolution training data

So far, our two-stage Kalman filter assumes the temporal resolution of the test set is identical to the training scan. To ensure our filter is generalizable, the following method was developed to reconstruct images acquired at a higher temporal resolution than that of the training data.

Suppose the temporal resolution of our test scan is five times that of our training scan, where the training scan represents frames 1, 6, 11 etc. in our test scan. The smallest phase transition in the test scan that our training scan can model is of size five (e.g. phase 1 to 6). To model smaller phase transitions while ensuring our estimate for $f({x}_{t-1}^{a})$ in Eq. (5) is accurate, a new procedure is necessary. As before, we use an affine transform to predict $f({x}_{t-1}^{a})$:

17 $${x}_{t}={f}_{t,new}{x}_{t-1}+{b}_{t,new},$$

where ${f}_{t,new}$ and ${b}_{t,new}$ are a state transition matrix and bias vector purposely designed to model a smaller phase transition.

However, as Eq. (17) is a model of cardiac dynamics, computation of ${f}_{t,new}$ and ${b}_{t,new}$ requires a priori information. While we cannot use the model learned in our cardiac motion dictionary, we can use the *information* in that dictionary to compute ${f}_{t,new}$ and ${b}_{t,new}$. Suppose the smallest possible phase transition our learned cardiac motion dictionary can model is that of size $V$. According to our procedure described above, we would model this phase transition by setting ${C}_{l}={C}_{1}$ in Eq. (14a). Thus, we have the following model for describing a transition from phase $t-V$ to phase $t$:

18 $${x}_{t}={f}_{V}{x}_{t-V}+{b}_{V}.$$

Let $t-v$ indicate an arbitrary phase in between phases $t-V$ and $t$, where $V-1\ge v\ge 1$. The cardiac phase immediately following $t-V$ in the test scan is $t-V+1$ (i.e. $v=V-1$). To reconstruct frame ${x}_{t-v}$, we assume this image falls linearly between ${x}_{t-V}$ and ${x}_{t}$:

19 $${x}_{t-v}=\frac{V-v}{V}\left({x}_{t}-{x}_{t-V}\right)+{x}_{t-V}.$$

When $v=V-1$, we are reconstructing the cardiac phase immediately following $t-V$. Substituting Eq. (18) into Eq. (19) yields:

20a $${x}_{t-v}=\frac{V-v}{V}\left({f}_{V}{x}_{t-V}+{b}_{V}-{x}_{t-V}\right)+{x}_{t-V},$$

20b $${x}_{t-v}=\left(\frac{V-v}{V}I{f}_{V}+\frac{v}{V}I\right){x}_{t-V}+\frac{V-v}{V}I{b}_{V}.$$

Taking the expected value of Eq. (20b) with respect to all past observations (${z}_{t-V}\dots$)^7,8,21^ yields the following:

21a $$E\left[{x}_{t-v}|{z}_{t-V}\dots \right]=\left(\frac{V-v}{V}I{f}_{V}+\frac{v}{V}I\right)E\left[{x}_{t-V}|{z}_{t-V}\dots \right]+\frac{V-v}{V}I{b}_{V},$$

21b $${x}_{t-v}^{f}=\left(\frac{V-v}{V}I{f}_{V}+\frac{v}{V}I\right){x}_{t-V}^{a}+\frac{V-v}{V}I{b}_{V}.$$

Equation (21b) is an affine transform that can be used to predict ${x}_{t-v}^{f}=f({x}_{t-V}^{a})$ using a previous fully reconstructed image (${x}_{t-V}^{a}$). While Eq. (21b) is valid if our goal is to reconstruct phase $t-v$ using phase $t-V$, it does not provide a solution to the problem of reconstructing phase $t-v+1$ from phase $t-v$. To generalize Eq. (21b), we rewrite Eq. (19) for phase $t-v+1$:

22 $${x}_{t-v+1}=\frac{V-v+1}{V}\left({x}_{t}-{x}_{t-V}\right)+{x}_{t-V}.$$

Substituting in Eq. (18) yields:

23a $${x}_{t-v+1}=\frac{V-v+1}{V}\left({f}_{V}{x}_{t-V}+{b}_{V}-{x}_{t-V}\right)+{x}_{t-V},$$

23b $${x}_{t-v+1}=\left(\frac{V-v+1}{V}I{f}_{V}+\frac{v-1}{V}I\right){x}_{t-V}+\frac{V-v+1}{V}I{b}_{V}.$$

Equation (20b) provides a model for ${x}_{t-V}$ in terms of ${x}_{t-v}$. Let us substitute Eq. (20b) into Eq. (23b):

24 $${x}_{t-v+1}=\left(\frac{V-v+1}{V}I{f}_{V}+\frac{v-1}{V}I\right){\left(\frac{V-v}{V}I{f}_{V}+\frac{v}{V}I\right)}^{-1}{x}_{t-v}-\left(\frac{V-v+1}{V}I{f}_{V}+\frac{v-1}{V}I\right){\left(\frac{V-v}{V}I{f}_{V}+\frac{v}{V}I\right)}^{-1}\frac{V-v}{V}{b}_{V}+\frac{V-v+1}{V}I{b}_{V}.$$

Taking the expected value of Eq. (24) with respect to all past observations (${z}_{t-v}\dots$) yields:

25a $${E[x}_{t-v+1}\left|{z}_{t-v}\dots \right]=\left(\frac{V-v+1}{V}I{f}_{V}+\frac{v-1}{V}I\right){\left(\frac{V-v}{V}I{f}_{V}+\frac{v}{V}I\right)}^{-1}E\left[{x}_{t-v}|{z}_{t-v}\right]-\left(\frac{V-v+1}{V}I{f}_{V}+\frac{v-1}{V}I\right){\left(\frac{V-v}{V}I{f}_{V}+\frac{v}{V}I\right)}^{-1}\frac{V-v}{V}{b}_{V}+\frac{V-v+1}{V}I{b}_{V},$$

25b $${x}_{t-v+1}^{f}=\left(\frac{V-v+1}{V}I{f}_{V}+\frac{v-1}{V}I\right){\left(\frac{V-v}{V}I{f}_{V}+\frac{v}{V}I\right)}^{-1}{x}_{t-v}^{a}-\left(\frac{V-v+1}{V}I{f}_{V}+\frac{v-1}{V}I\right){\left(\frac{V-v}{V}I{f}_{V}+\frac{v}{V}I\right)}^{-1}\frac{V-v}{V}{b}_{V}+\frac{V-v+1}{V}I{b}_{V}.$$

Equation (25b) is an affine transform capable of predicting the subsequent cardiac phase $t-v+1$ using the fully reconstructed image for phase $t-v$. Thus, for our new model defined by Eq. (17):

26a $${f}_{t,new}=\left(\frac{V-v+1}{V}I{f}_{V}+\frac{v-1}{V}I\right){\left(\frac{V-v}{V}I{f}_{V}+\frac{v}{V}I\right)}^{-1},$$

26b $${b}_{t,new}=\left(\frac{V-v+1}{V}I{f}_{V}+\frac{v-1}{V}I\right){\left(\frac{V-v}{V}I{f}_{V}+\frac{v}{V}I\right)}^{-1}\frac{V-v}{V}{b}_{V}+\frac{V-v+1}{V}I{b}_{V}.$$

To reiterate: $V$ describes the smallest phase transition our learned cardiac motion dictionary can model, ${f}_{V}$ and ${b}_{V}$ are the associated state transition matrix and bias vector from the learned cardiac motion dictionary, and $v$ is an indexing variable to help the user track which cardiac phase of the test scan between phases $t-V$ and $t$ is being reconstructed.

Equations (26a) and (26b) provide a method for computing the appropriate state transition matrix and bias vector in Eq. (17) despite a low temporal resolution training scan. By enabling reconstruction at smaller phase increments, we are effectively reconstructing at an enhanced temporal resolution. Furthermore, as these equations were derived for an arbitrary value of $V$, we may generalize $V$ to represent any phase transition that can be adequately modeled by our learned cardiac motion dictionary. Thus, Eqs. (26a) and (26b) enable us to reconstruct multiple cardiac cycles, arrhythmic events, or changes in the sinus rhythm (as ${f}_{V}$ and ${b}_{V}$ are known), further generalizing our predictive signal model and ensuring it is adaptable to any sinus rhythm no matter how different the true rhythm is from our training data.

#### Initializing R, ${\mathrm{x}}_{0}^{\mathrm{a}}$, and ${\mathrm{P}}_{0}^{\mathrm{a}}$

Given the orthogonality of *k-*space data points, one can assume that *R* is additive white Gaussian noise. Hence, $R={\sigma }^{2}I$, where ${\sigma }^{2}$ is the variance of k*-*space corresponding to an arbitrarily selected background region within any test image. Ideally, *R* would be estimated via a phantom scan. The initial value for the reconstructed image (${x}_{0}^{a}$) is chosen to be the first image in the training set, as any image can be used to initialize the Kalman filter^9,11^. For the first time-step only, this requires ${f}_{1}={I}_{{N}^{2}\times {N}^{2}}$. To initialize our spatiotemporal covariance (${P}_{0}^{a}$), we initially assume that image noise is additive white Gaussian. As such, ${P}_{0}^{a}$ is a diagonal matrix, where the diagonal entries of ${P}_{0}^{a}$ are equal to the spatial variance of an arbitrarily selected background segment within any test image. In the majority of our simulations, this background corresponds to the one used to estimate $R$; raw *k*-space noise was used instead if applicable. It is important to note that this estimate of ${P}_{0}^{a}$ may not be accurate, as it captures the *spatial* statistics of our image. However, the recursive updates of the Kalman filter correct for these inaccuracies, ensuring the *spatiotemporal* statistics are properly represented by ${P}_{t}^{a}$.

#### Implementation of the random-walk Kalman filter

In order to test the effectiveness of our predictive signal model, we designed a random-walk Kalman filter based on the state-space model used in previous work. Our implementation of the random-walk Kalman filter is not identical to prior work, as it does not consider supplementary techniques.

In our random-walk Kalman filter, $Q$ was computed in two steps. First, the training scan was used to compute a sequence of *T-1* difference images. Second, the covariance of each pixel in the difference images was computed and assigned to the corresponding diagonal entry of $Q$. This procedure was inspired by previous work, where the off-diagonal entries of $Q$ were assumed to be of low power and ignored. Aside from $f\left({x}_{t-1}^{a}\right)= {x}_{t-1}^{a}$ (which implies ${J}_{f}\left({x}_{t-1}^{a}\right)={I}_{{N}^{2}\times {N}^{2}}$), all other parameters of the Kalman filter were unaltered.

### Compressed sensing implementation

We will now discuss how to integrate the predictive signal model with a GRASP CS scheme. Let us begin with the following minimization problem:

27 $$\widehat{X}=\underset{X}{\mathrm{argmin}}{\Vert HX-y\Vert }_{2}^{2}+{\lambda }_{spar}\varphi \left(X\right)+{\lambda }_{ss}{\sum }_{m=2}^{M}{\Vert {f}_{m}X{A}_{m-1}+{b}_{m}-X{A}_{m}\Vert }_{2}^{2},$$

where $X$ is a matrix with each column representing a single fully sampled image (organized temporally), $\widehat{X}$ represents the final reconstructed images, $y$ is the acquired *k-*space data, $H$ is a measurement model relating $X$ to $y$, $\varphi ()$ is a sparsity transform, ${f}_{m}$ and ${b}_{m}$ are the state transition matrix and bias vector that model a phase transition from time point $m$ to $m+1$, and ${A}_{m}$ is a matrix to extract the *m*th column from $X$. We will refer to the last term as the state-space consistency term, as it ensures the reconstruction follows our predictive signal model. The parameters ${\lambda }_{spar}$ and ${\lambda }_{ss}$ are used to weigh the importance of the sparsity and state-space consistency terms during the reconstruction process.

The procedure for computing ${f}_{m}$ and ${b}_{m}$ remains unchanged: one must simply acquire a fully sampled training scan consisting of a single cardiac cycle. One may also account for low temporal resolution training data using the method derived in Eqs. (17)–(26b). Aside from weighting, the purpose of ${\lambda }_{ss}$ is to accommodate for inaccuracies and redundancies in the training data, thereby ensuring the predictive signal model generalizes well. Lastly, the state-space consistency term can be removed from Eq. (27) by setting ${\lambda }_{ss}=0$. This enables us to directly examine the impact of the predictive signal model on the CS reconstructions. Going forward, we will refer to CS with the predictive signal model as “two-stage CS”.

## Methods

Please note that all reconstructions in this paper were performed in MATLAB version R2020a or later.

This study consists of simulations that utilize DICOM cardiac CINE datasets from the UK Biobank and publicly available raw cardiac *k-*space datasets from the Ohio State University, denoted OCMR^25^. Relevant guidelines and regulations as specified by the UK Biobank were strictly adhered to. Our usage of UK Biobank data was approved via a Material Transfer Agreement (MTA) between our laboratory and the UK Biobank. The Innovations and Partnerships Office at the University of Toronto helped us execute said agreement. The application reference number for this MTA is 61943. Thus, this MTA serves as an ethics approval for a collaboration between our laboratory and the UK Biobank. With this MTA in place, the UK Biobank consents to the Data Protection Act of 1998. Informed consent was obtained by the UK Biobank for all patient data found within their repository, and there is no patient identifying information in the data provided to us. More information regarding the UK Biobank may be found in the data accessibility statement. For the OCMR datasets, all relevant guidelines and regulations specified by the Ohio State University for public use of the OCMR datasets were strictly adhered to. The regulations set forth by the Ohio State University explicitly state that no patient identifying information is included in the OCMR datasets. More information regarding the OCMR datasets may be found in the data availability statement.

To examine the performance of our predictive signal model, multiple datasets from the UK Biobank were used. To facilitate comparison across all simulated scenarios, reconstructions using UK Biobank data were performed with Kalman filtering only. A total of five different patient datasets from the UK Biobank were used for our simulations (previously acquired on a 1.5 T scanner (MAGNETOM Aera 1.5 T, Siemens Healthineers)). Each patient’s dataset consisted of two CINE scans, each representing a distinct fully sampled cardiac cycle with 50 phases. The first cardiac cycle was used for training. The second cycle was replicated five times to create ground truth data for testing, after which undersampling was performed to test reconstruction via Kalman filtering. Relevant parameters for each dataset are listed in Table 1. In total, three primary scenarios and two secondary scenarios simulating low temporal resolution training data were tested. All UK Biobank reconstructions were performed without using rebinning strategies common in conventional CINE. Data was assessed using mean squared error (MSE) and confirming MSE convergence.

**Table 1:** Relevant parameters for each dataset. It assumed that one TR corresponds to one phase encode/radial spoke.

	Dataset 1	Dataset 2	Dataset 3	Dataset 4	Dataset 5	OCMR Dataset
Cardiac view	Two-chamber long axis	Four-chamber view	Two-chamber long axis	Two-chamber long axis	Four-chamber view	Four-chamber view
Image size	64 × 64	70 × 70	60 × 60	60 × 60	70 × 70	136 × 81
Training time: primary scenarios	591 s	908 s	482 s	476 s	888 s	4333 s
Training time: secondary scenarios	572 s	N/A	N/A	N/A	N/A	N/A
Number of samples: cartesian	64	70	60	60	70	136
Number of phase encodes: cartesian	5	5	5	5	5	6–13
Acceleration rate: cartesian	$\left(\frac{64}{64}\right)\left(\frac{64}{5}\right)=12.8$	$\left(\frac{70}{70}\right)\left(\frac{70}{5}\right)=14$	$\left(\frac{60}{60}\right)\left(\frac{60}{5}\right)=12$	$\left(\frac{60}{60}\right)\left(\frac{60}{5}\right)=12$	$\left(\frac{70}{70}\right)\left(\frac{70}{5}\right)=14$	$\left(\frac{136}{136}\right)\left(\frac{81}{9.16}\right)=8.83$
Number of samples: radial	82	90	77	77	90	N/A
Number of spokes: radial	5	6	5	5	6	N/A
Acceleration rate: radial	20.1	18.3	18.9	18.9	18.3	N/A

To create undersampled test CINE scans from the UK Biobank data, full resolution images from the second cardiac cycle were converted to *k*-space via the 2D Fourier transform. An undersampling mask was applied and a NUFFT used for non-Cartesian trajectories. Undersampling masks and the NUFFT were designed to sample approximately 8% of *Cartesian k*-space for each frame (note that the NUFFT is not a one-to-one operation). Thus, each frame was undersampled by a factor of approximately 12.5. In our work, the undersampling factor can be viewed as an estimate of the acceleration relative to a fully sampled CINE sequence, especially for cartesian acquisition schemes. However, one must remember that the undersampling factor is not indicative of the true acceleration factor, as acceleration depends on multiple parameters such as the repetition time (TR) and the number of *k-*space lines acquired per frame (e.g. radial spokes). Table 1 provides a more tangible estimate of acceleration. Acceleration for radial scans was estimated using $\pi n/2{n}_{s}$, where $n$ is the number of points in the fully reconstructed image, and ${n}_{s}$ is the number of acquired spokes. Note: it is conventional to acquire $\pi n/2$ spokes during radial acquisition^26^. This formula also assumes a constant TR and oversampling ratio between a conventional radial scan and our accelerated radial scan.

To verify the performance of our algorithms on raw *k-*space data, a dataset from the OCMR library was used (previously acquired on a 1.5 T scanner (MAGNETOM Sola 1.5 T, Siemens)). Reconstructions were performed using both Kalman filtering and CS. The filename for this dataset was “us_209_pt_1_5T.h5”. This dataset consisted of prospectively undersampled Cartesian *k-*space acquired over multiple cardiac cycles. To reduce the field of view and provide better visualization of the heart, every other sample along the frequency and phase encode direction was taken; aliasing was removed in post processing. To create the training set, this dataset was rebinned into a fully sampled 15 phase cardiac CINE. After building the learned cardiac motion dictionary, reconstruction of 30 unsorted prospectively undersampled raw *k-*space frames was performed. Each frame sampled approximately 11.32% of Cartesian *k*-space, which corresponds to an undersampling factor of approximately 8.83. Note that for the OCMR dataset only, the sampling mask changed between successive frames. The 30 frames were chosen to simulate two *distinct* periodic cardiac cycles, thereby providing assurance that our algorithms can reconstruct multiple cardiac cycles acquired from raw *k*-space data. Reconstruction parameters are listed in Table 1. In total, one primary scenario was tested. The OCMR dataset consists of raw *k-*space acquired from multiple receiver coils. However, our experiments only used raw *k*-space acquired from one of these receiver coils. This was done to fully demonstrate the capabilities of our predictive signal model in the absence of any supplementary techniques.

### Primary scenarios: algorithm feasibility

The three primary scenarios were designed to evaluate the performance of our predictive signal model, assuming identical temporal resolution in the training and test data. To evaluate the feasibility and robustness of our predictive signal model, each primary scenario was repeated across all five UK Biobank datasets. For each UK Biobank simulation, the two-stage Kalman filter was compared to the random-walk Kalman filter. To verify that our predictive signal model can facilitate raw *k-*space data reconstruction, the first primary scenario was repeated an additional time using the OCMR dataset. For each OCMR simulation, the two-stage Kalman filter and the two-stage CS scheme were compared to the random-walk Kalman filter and a conventional CS scheme. Conventional CS was achieved by setting ${\lambda }_{ss}=0$ in Eq. (27).

In scenario one for the UK Biobank datasets, we reconstructed a test set with the same heart rate as the training set. The training set consisted of one cardiac cycle with $T=50$ phases. These images were denoised in MATLAB using a built-in convolutional neural network (cNN). The Moore–Penrose pseudo-inverse was used to ensure successful computation of Eq. (14a). To generate the test set, the second cardiac cycle (i.e. test set) from the databank was replicated 5 times, creating $M=5T=250$ images for five periodic cardiac cycles. To handle a phase transition from one heartbeat to the next, we transition from phase 50 to phase 1 of the next heartbeat, implying $l=- 49$. To protect against contrast variations due to automated rescaling of DICOM images, each frame in the test set was rescaled to match the mean of the corresponding phase in the training set. This scenario tests our predictive signal model under theoretically ideal conditions, as periodicity was vital to previous work.

In scenario one for the OCMR dataset, we reconstructed 30 frames of raw *k*-space data. The training set consisted of one cardiac cycle with $T=15$ phases. These training images were not denoised in MATLAB, as we wished to preserve the integrity of the raw *k*-space. As all *k-*space was acquired from a single experiment, the mean of each frame in the test set was not rescaled. For our CS schemes, we used a non-linear conjugate gradient descent algorithm based on backtracking line search to solve Eq. (27). We also used temporal total variation (TTV) as our sparsity transform $\varphi ()$.

In scenario two for the UK Biobank datasets, we simulated arrhythmia by creating an early return to systole. In the 50-phase cardiac cycle from the biobank, phase 1 corresponds to early systole synchronized with the R-wave of the ECG signal and phase 50 corresponds to the end of diastole. To mimic an early occurrence of the next R-wave, we made the simple assumption that the heart was beating to phase 30 and then jumped to phase 1 of the next cycle. To recreate a shorter cycle for the second of the 5 consecutive cycles in the test set, only phases 1–30 were retained; the other four cycles used all 50 phases. Effectively, we transitioned from phase 30 to 1, implying $l=-29$. The mean of each frame in the test set was rescaled.

In scenario three for the UK Biobank datasets, we simulate a transient change in heart rate (faster sinus rhythm). For simplicity, we assumed the heart rate doubled during the second of the 5 consecutive cycles in the test set. For this second cardiac cycle, every other phase was retained (i.e. phase 1, 3, 5,…), implying $l=2$. All other cycles consisted of 50 phases. The mean of each frame in the test set was rescaled.

To demonstrate the flexibility of our predictive signal model, each UK Biobank simulation was performed two separate times using Cartesian and golden-angle radial undersampling masks (all scenarios using the first UK Biobank dataset were repeated with a spiral undersampling mask as well). All undersampling for Cartesian masks was performed in the phase encode direction. The OCMR simulations were performed using a Cartesian mask included in the OCMR dataset. All these masks were tested in order to demonstrate our signal model’s ability to handle versatile undersampling schemes. To facilitate spiral acquisition, a total of 6 rotations were used. The sampling scheme used in the OCMR datasets was similar to variable density Cartesian, and was left unchanged. To accommodate for non-Cartesian trajectories, a NUFFT based on GRASP was used for $EF$^18^. All relevant parameters are found in Table 1; an acceleration rate estimate is not provided for spiral undersampling, as the acquisition time per frame is equal to TR.

### Secondary scenario: accommodating for low temporal resolution training data

In two different scenarios, we evaluated the performance of the two-stage Kalman filter against the random-walk Kalman filter, assuming the temporal resolution of the test set was five times that of the training set. The training and test sets were obtained from the first UK Biobank dataset.

In our first secondary scenario, the training set consisted of one cardiac cycle with $T=10$ phases. These images were denoised in MATLAB, and the Moore–Penrose pseudoinverse was used to ensure successful computation of Eq. (14a). The test set consisted of five periodic cardiac cycles, with 50 phases per cycle. The mean of each frame in the test set was also rescaled. As certain test set images did not have a corresponding training set image, their means were scaled to match the mean of the previous image in the test set. Reconstruction was performed using a radial mask. A NUFFT based on GRASP was also used here.

An additional secondary scenario was simulated using a modified state-space model where ${f}_{t}={I}_{{N}^{2}\times {N}^{2}}$, ${b}_{t}=0$ and $Q={C}_{0}$. This simulation is analogous to a random-walk Kalman filter, except $Q$ has off-diagonal entries. The purpose of this simulation was to discern the importance of the off-diagonal entries in ${f}_{t}$ and their ability to control the high power of $Q$, particularly if reconstruction is performed using a low temporal resolution training set.

## Results

Reconstruction times for all scenarios can be found in Table 2. Reconstruction times for the CS schemes were not reported, as each reconstruction completed in under 1 min. A comparison against Kalman filter reconstruction times would be insignificant.

**Table 2:** Reconstruction times (s) for all scenarios. Note, “D2” stands for “Dataset 2” etc.

	Primary scenarios: algorithm feasibility			Secondary scenario	
	Scenario 1: multiple periodic cycles	Scenario 2: single arrhythmic event	Scenario 3: faster sinus rhythm	Scenario 1: two-stage Kalman filter	Scenario 2: $f={I}_{{N}^{2}\times {N}^{2}}, b=0,$ non-diagonal process covariance
Two-stage Kalman filter, Cartesian sampling	D1: 2610	D1: 1950	D1: 1961	N/A	N/A
	D2: 4180	D2: 2976	D2: 3152		
	D3: 1689	D3: 1270	D3: 1448		
	D4: 1698	D4: 1461	D4: 1188		
	D5: 4274	D5: 3188	D5: 3051		
Two-stage Kalman filter, radial sampling	D1: 2601	D1: 1911	D1: 1879	2682	1905
	D2: 4523	D2: 3185	D2: 3245		
	D3: 1780	D3: 1339	D3: 1317		
	D4: 1726	D4: 1322	D4: 1267		
	D5: 4300	D5: 3305	D5: 3182		
Two-stage Kalman filter, spiral sampling	D1: 2685	D1: 2027	D1: 2016	N/A	N/A
Random-walk Kalman filter, cartesian sampling	D1: 1830	D1: 1730	D1: 1669	N/A	N/A
	D2: 3026	D2: 2690	D2: 2645		
	D3: 1267	D3: 1137	D3: 1097		
	D4: 1223	D4: 1105	D4: 1082		
	D5: 3140	D5: 2818	D5: 2752		
Random-walk Kalman filter, radial sampling	D1: 1907	D1: 1739	D1: 1743	1892	
	D2: 3210	D2: 2921	D2: 2911		
	D3: 1273	D3: 1166	D3: 1176		
	D4: 1318	D4: 1365	D4: 1138		
	D5: 3354	D5: 3028	D5: 3011		
OCMR dataset, two-stage Kalman filter	5980	N/A	N/A	N/A	
OCMR dataset, Random-walk Kalman filter	3969	N/A	N/A	N/A	

### Primary scenarios: algorithm feasibility

**Figure 2:**
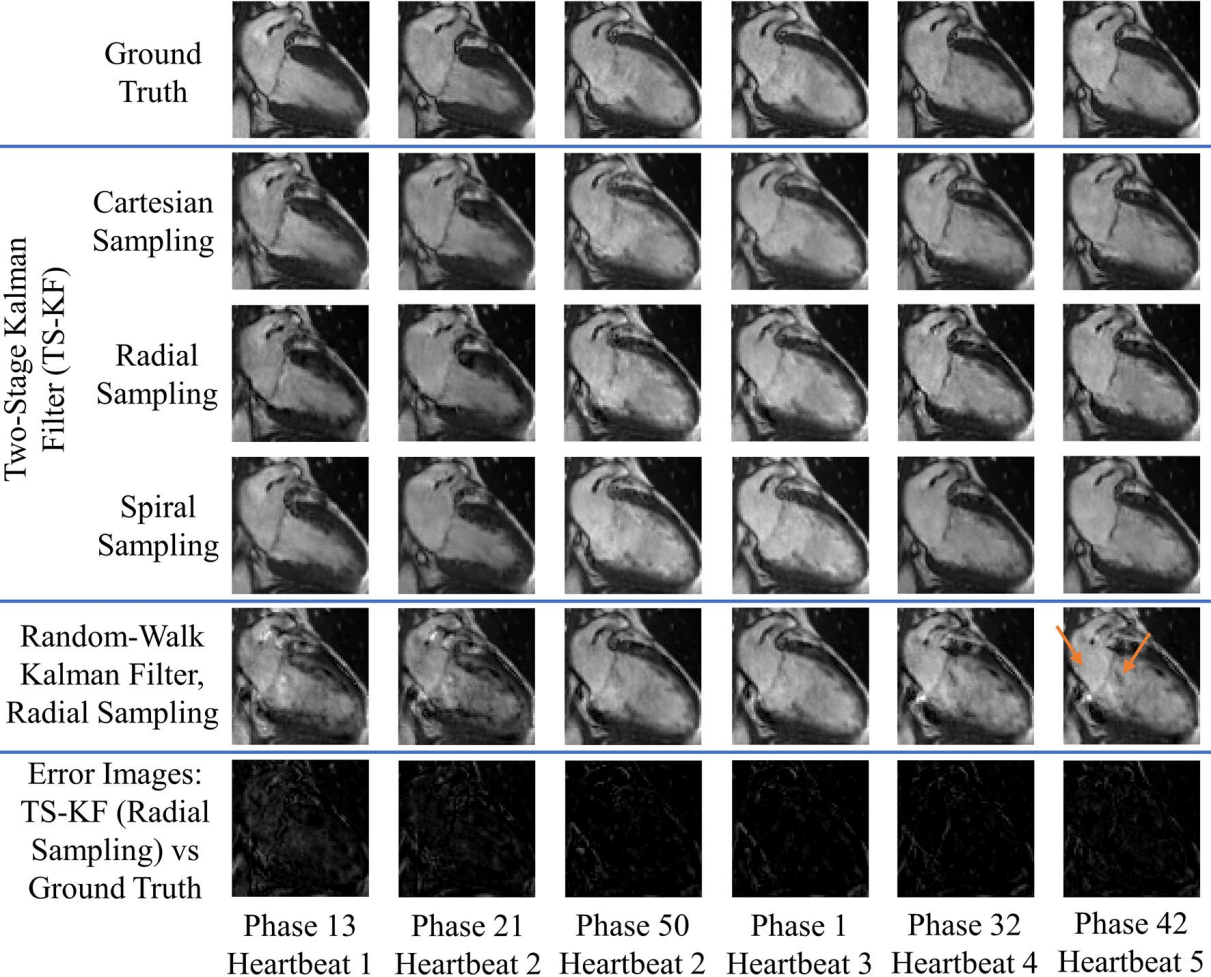
Reconstruction of multiple cardiac cycles I. These reconstructions were performed on the first UK Biobank dataset. The orange arrows illustrate distortions in the shape of the left atrium and the contrast along the wall of the left ventricle during random-walk Kalman filter reconstructions. Our two-stage Kalman filter demonstrates improved tracking and reconstruction quality compared to the random-walk Kalman filter.

**Figure 3:**
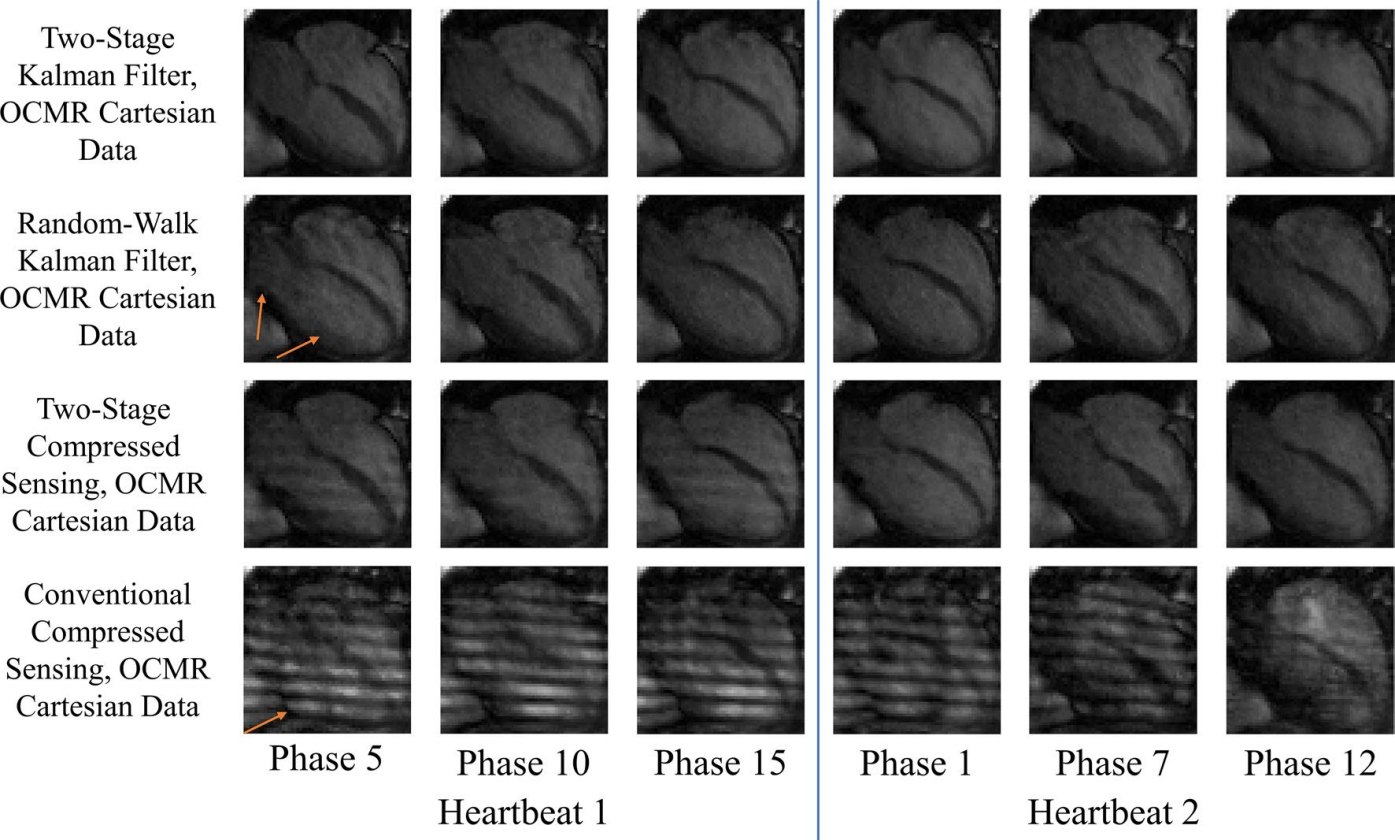
Reconstruction of multiple cardiac cycles II. These reconstructions were performed on the prospectively undersampled OCMR dataset. The orange arrows illustrate the inability of the random-walk Kalman filter and conventional CS to properly model cardiac dynamics for the given undersampling scheme. Equipped with a predictive signal model, both our two-stage Kalman filter and two-stage CS scheme demonstrate improved tracking and reconstruction quality compared to the random-walk Kalman filter and conventional CS when applied to raw *k*-space datasets.

**Figure 4:**
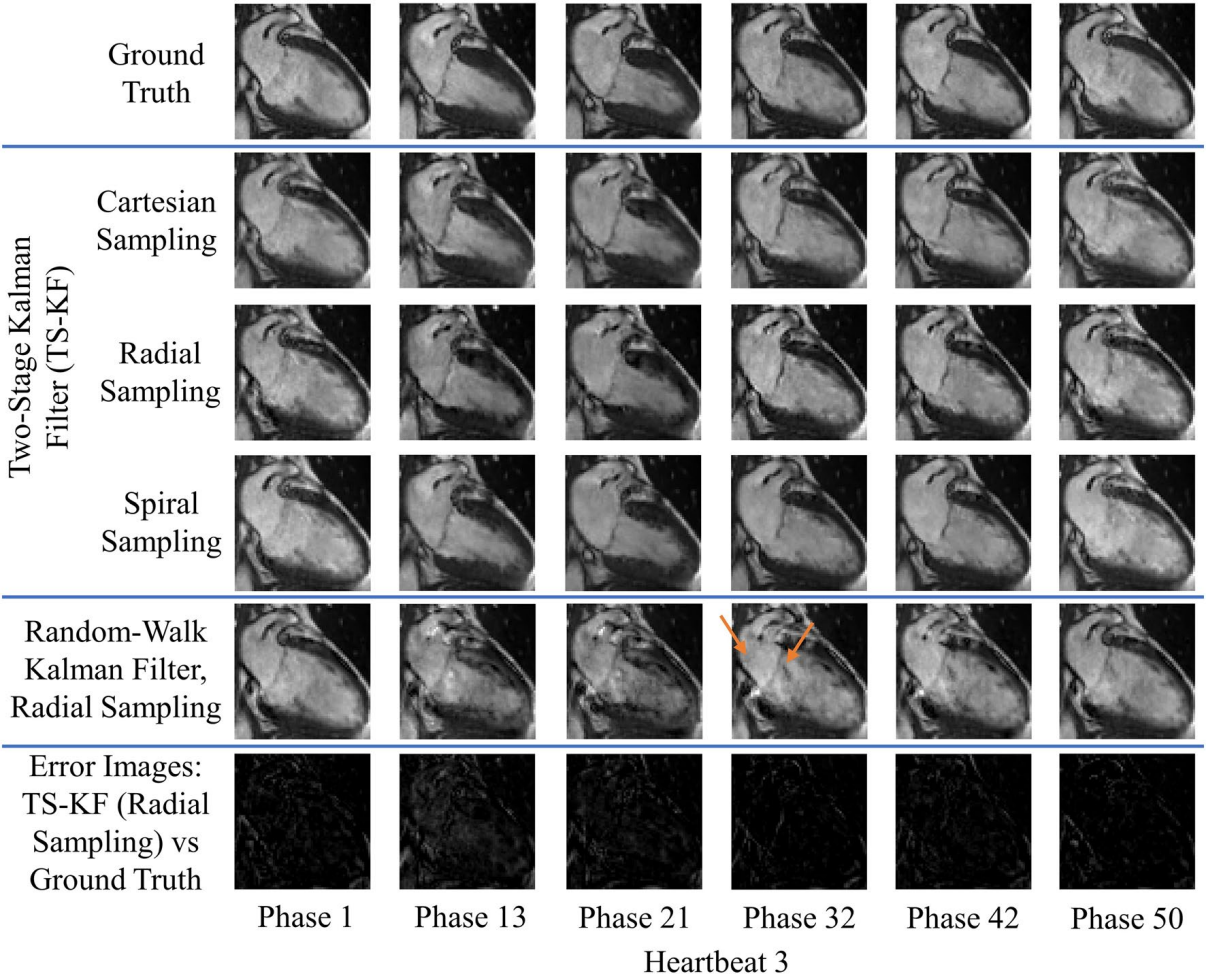
Reconstruction of a single arrhythmic event. These reconstructions were performed on the first UK Biobank dataset. Arrhythmia is simulated as skipping phases 8 and 9 in heartbeat 2 and a sudden return to systole (phase 1) in heartbeat 3. The orange arrows illustrate distortions in the shape of the left atrium and the contrast along the wall of the left ventricle during random-walk Kalman filter reconstructions. The two-stage Kalman filter can track the arrhythmic event and continuously provide high-quality reconstructions afterwards.

**Figure 5:**
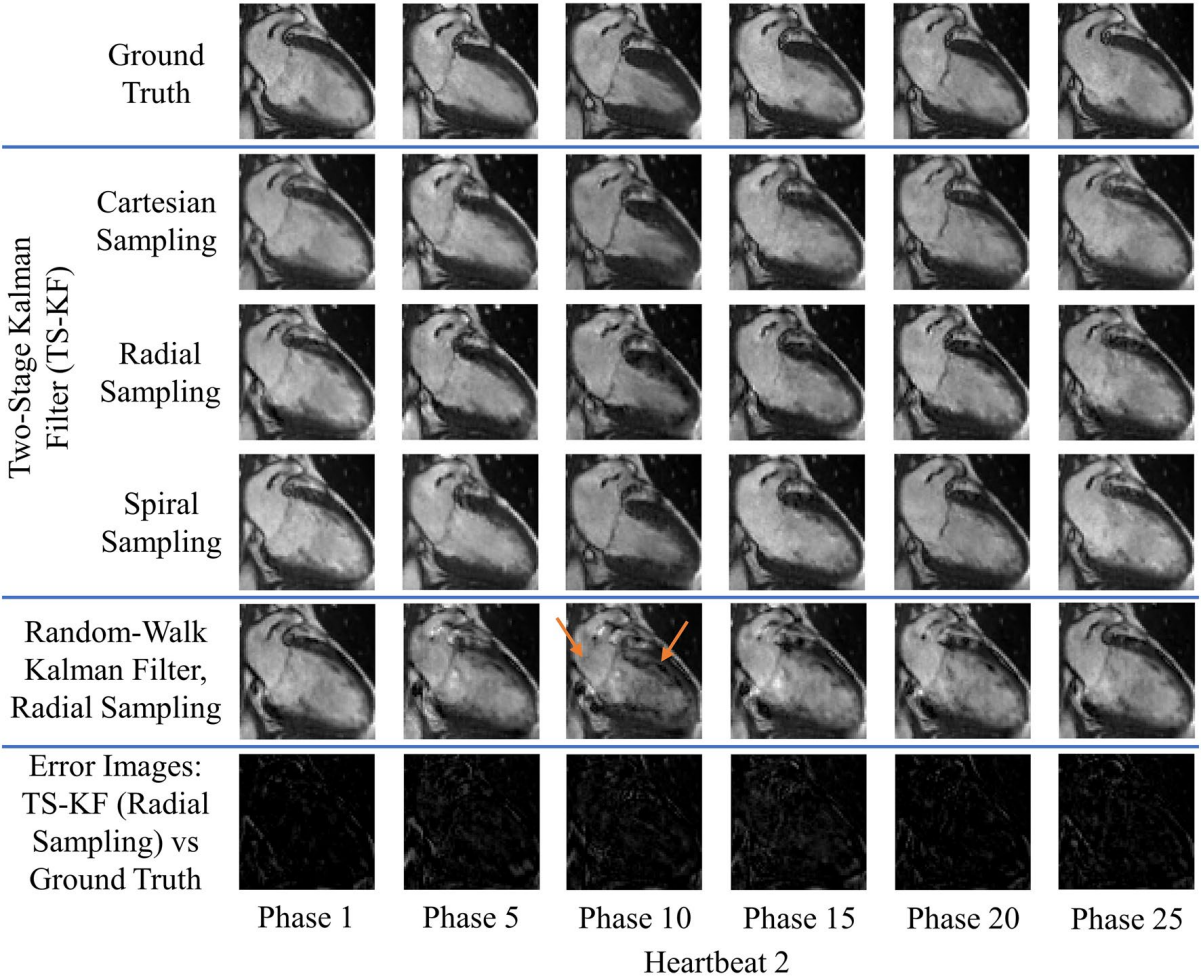
Reconstruction in the presence of variable sinus rhythm. These reconstructions were performed on the first UK Biobank dataset. A doubling of the sinus rhythm is simulated by skipping every other phase in heartbeat 2. The orange arrows demonstrate perturbations in the shape of the left atrium and the contrast along the wall of the left ventricle during random-walk Kalman filter reconstructions. Our two-stage Kalman filter demonstrates improved tracking and reconstruction quality compared to the random-walk Kalman filter.

**Figure 6:**
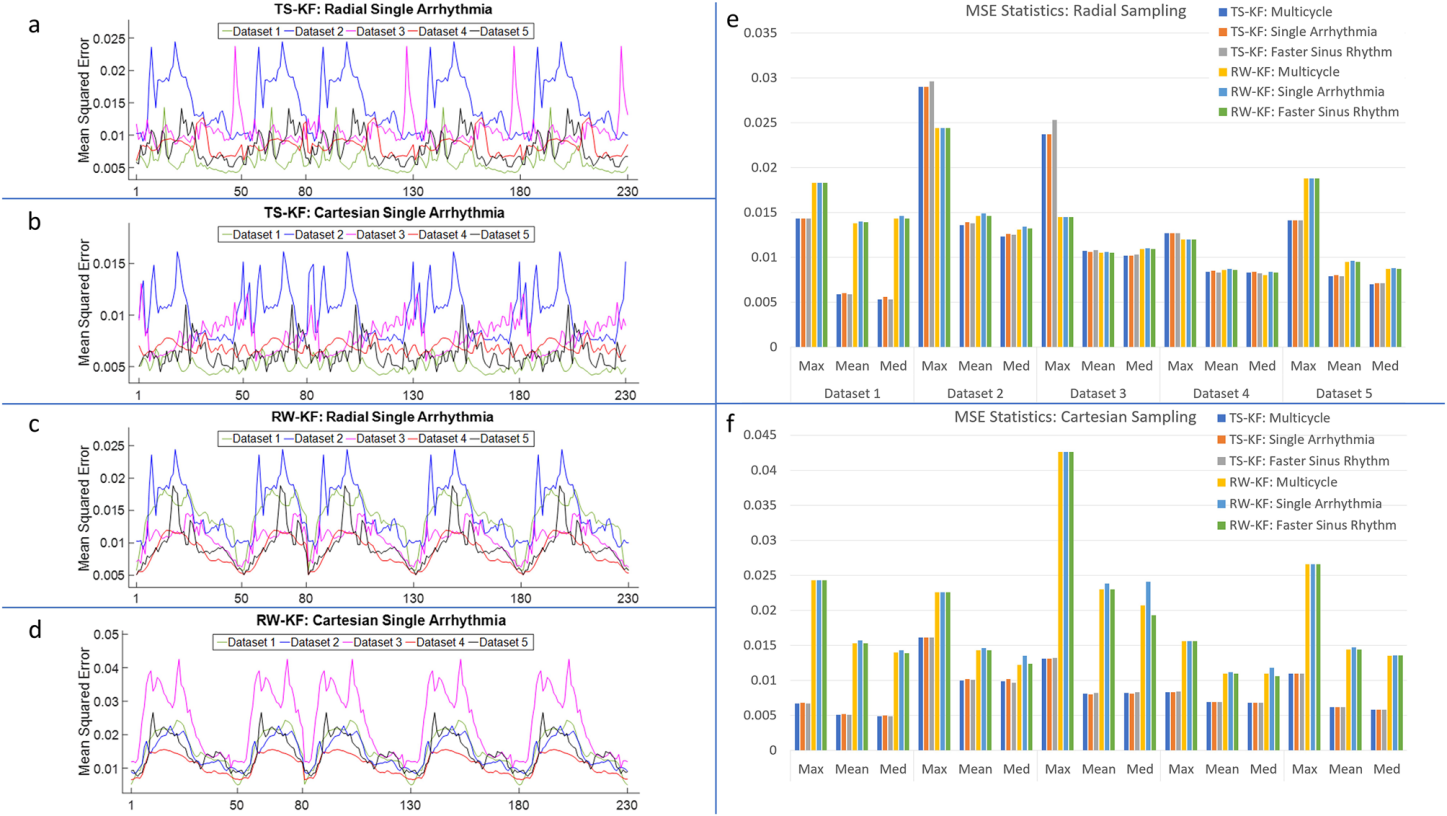
Mean-squared error (MSE) plots for the second primary scenario (single arrhythmic event), and MSE statistics for all scenarios. The subplots demonstrate the MSE for the second primary scenario across all UK Biobank datasets: (**a**) two-stage Kalman filter (TS-KF) with radial sampling, (**b**) TS-KF with cartesian sampling, (**c**) random-walk Kalman filter (RW-KF) with radial sampling, and (**d**) RW-KF with cartesian sampling. The x indices represent the frame number. Except for the index labeled “1”, each index also indicates the last phase of the corresponding cardiac cycle. (**a–d**) All demonstrate the robustness and consistency of our two-stage Kalman filter. Across all datasets, the MSE exhibited convergence. Subplot (**e**) demonstrates the maximum, mean, and median (med) values for all primary scenarios using radial sampling across all UK Biobank datasets. The MSE for the OCMR dataset is not available, as prospectively undersampling precludes the availability of the ground truth. Note that in general the TS-KF offered improved or comparable performance to the RW-KF. Subplot (**f**) demonstrates the maximum, mean, and median values for all primary scenarios using cartesian sampling across all datasets. Note that the TS-KF vastly outperforms the RW-KF for this sampling scheme.

Figures 2, 3, 4 and 5 demonstrate improved tracking and reconstruction of the two-stage Kalman filter and the two-stage CS scheme. The ability to successfully track dynamics across multiple cardiac cycles (Figs. 2, 3), after unforeseen changes in cardiac dynamics (Fig. 4), and during changes in the heart rate (Fig. 5) are shown. The MSE results for the second primary scenario (single arrhythmia) are shown in Fig. 6a–d. This scenario was chosen due to the increased difficulty of modelling an arrhythmic event. The MSE results for the first and third primary scenarios are shown in Supplementary Figs. S1 and S2. The MSE for the OCMR dataset cannot be provided, as the usage of prospectively undersampled data precludes the availability of a ground truth. All calculated MSE results displayed convergence, thereby demonstrating the feasibility and robustness of our predictive signal model. Figure 6e,f displays the maximum, mean, and median MSE values for all radial and cartesian simulations. Interestingly, the random-walk Kalman filter offered comparable MSE statistics to the two-stage Kalman filter for specific datasets using radial sampling (Fig. 6e). However, for Cartesian sampling MSE statistics (Fig. 6f), the two-stage Kalman filter demonstrated superior performance compared to the random-walk Kalman filter.

Both the random-walk Kalman filter and conventional CS introduced distortions in the reconstructed images. For the random-walk Kalman filter, the shape of the left atrium and the contrast along the wall of the left ventricle was affected (see arrows in Figs. 2, 3, 4, 5). As an example, consider the OCMR random-walk Kalman filter reconstructions: the left atrium and left ventricle are improperly shaped for a contracting heart (see orange arrows). These distortions were observed to occur even if the random-walk Kalman filter occasionally produced a reconstruction of sufficient quality (e.g. Fig. 4, heartbeat 3, phases 1 and 50). In regards to conventional CS, streaking artifacts were observed throughout the entire image. This is consistent with insufficient sampling density in the phase encode direction and the usage of Cartesian undersampling.

Overall, an increase in image quality and convergence of the MSE (if applicable) was observed for all simulations. Figures 2, 3, 4 and 5 demonstrate that radial sampling was the best undersampling scheme for our algorithms, despite absence of a lower MSE.

### Secondary scenario: accommodating for low temporal resolution training data

**Figure 7:**
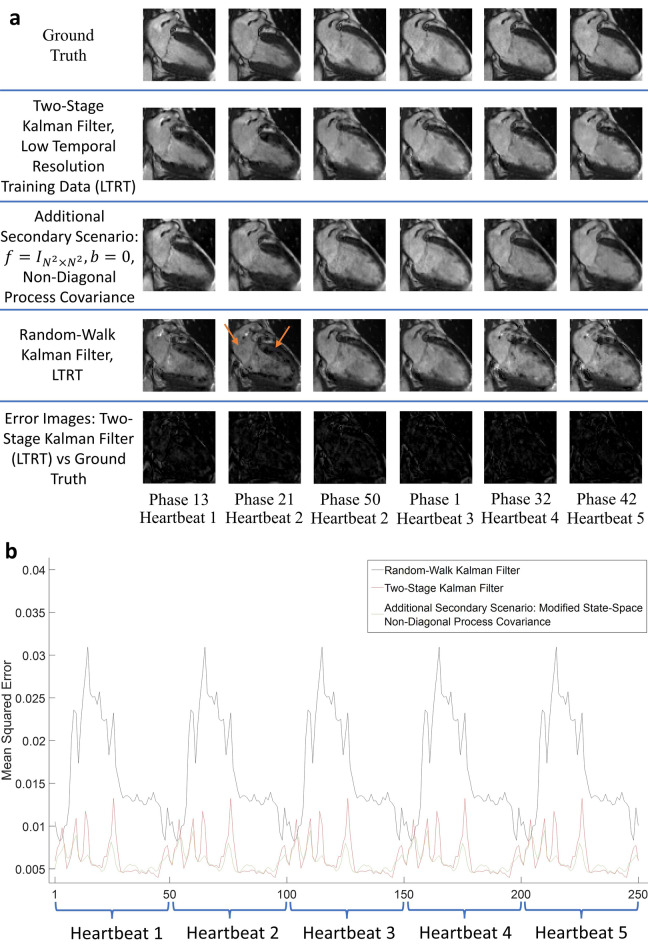
Reconstruction accommodating for low temporal resolution training data. All reconstruction results are shown in (**a**). The test set has a temporal resolution five times greater than that of the training set. Combined with a spatial undersampling factor of 12.5, the effective acceleration factor is 100.5. Note that the random-walk Kalman filter reconstructs erroneous images: this is a consequence of inaccurate modelling. To compensate, the Kalman filter continuously attempts to reconstruct the mean, hence the perturbations. These perturbations are highlighted with orange arrows. Subplot (**b**) provides the MSE for the reconstructions shown in (**a**). The MSE plot for our two-stage Kalman filter simulation is shown in red. Our algorithm properly accommodates low temporal resolution training data by ensuring convergent behavior. Furthermore, the magnitude of the error is comparable to those shown in Fig. 6.

A total of two hundred and fifty $64\times 64$ images were reconstructed for both secondary scenarios (Fig. 7a). The MSE results for these simulations are shown to converge in Fig. 7b. A video of the reconstructions for our two-stage Kalman filter with a 5$\times$ increase in temporal resolution can be found as Supplementary Video S2. These results demonstrate the ability of our predictive signal model to reconstruct cardiac images across multiple cardiac cycles despite low temporal resolution training data. Notice that in Fig. 7a significant noise appears in the random-walk Kalman filter reconstructions, as indicated by the arrows. This is a consequence of inaccurate cardiac modelling, demonstrating that the predictive signal model is necessary to achieve robust dynamic CMR.

In Fig. 7b, note that setting ${f}_{t}={I}_{{N}^{2}\times {N}^{2}}$, ${b}_{t}=0$, and $Q={C}_{0}$ caused the MSE to exhibit divergent trends. These divergent trends were found by examining how the variance of the MSE changes with time. Interestingly, this simulation yielded a reconstruction quality close to our two-stage Kalman filter (Fig. 7a). Taken together, this suggests that the off-diagonal terms used to compute ${f}_{t}$ and ${b}_{t}$ are necessary. Moreover, a predictive signal model *must* be used to ensure convergence if the training data has a lower temporal resolution than that of the test set. This justifies the need for a large matrix ${f}_{t}$, and proves the ability of ${f}_{t}$ to control the large power of $Q$ via smoothing. As such, the off-diagonal terms in ${f}_{t}$ contribute substantially to successful image reconstruction.

## Discussion

Our algorithms provided improved reconstruction quality at an average undersampling factor of 12.5 across all sampling masks while demonstrating convergence. Furthermore, our algorithms demonstrated improved performance when applied to raw *k-*space data. Most importantly, our predictive signal model is potentially amenable to a plethora of scenarios in dynamic CMR and may provide significant advantages in the form of: adaptability to irregular cardiac rhythm, versatile undersampling, opportunity to compensate for low temporal resolution training data, and the ability to be integrated with previously developed reconstruction techniques.

While our results are promising, it must be emphasized that our work was largely based on simulations, as raw *k*-space data in all the formats we desired (non-Cartesian k-space, arrhythmia, variable sinus rhythm) was unavailable. In fact, this relative dearth of ground-truth cardiac k-space data has been a long-standing issue and is the reason why all real-time cardiac MRI methods have been notoriously difficult to verify. Despite this limitation, we did demonstrate the robustness of our algorithms when applied to raw Cartesian k-space cardiac data that had been prospectively undersampled. Other undersampled k-space trajectories, such as radial and spiral, had to be simulated from Cartesian DICOM images, which is non-ideal because artifacts commonly associated with radial/spiral imaging, as well as strategies to combat these artifacts (e.g. density compensation and trajectory correction), were absent. Furthermore, abnormal cardiac dynamics, such as arrhythmia and changing heart rate, are not found in real data and had to be simulated. The reasons are obvious, as conventional re-binning of acquired frames is intended to eliminate abnormal cardiac dynamics; even if there exists prospectively acquired data on an abnormal rhythm, there is no means to verify it.

Amongst the three undersampling schemes, the improved performance offered by radial sampling is not surprising, as this approach emphasizes the low frequency region of *k*-space; hence, one would expect improved reconstruction of global structural details. However, the two-stage Kalman filter MSE from radial sampling occasionally exhibited comparable behavior to the random-walk Kalman filter MSE. Furthermore, the MSE for both Kalman filters displayed the occasional spike, which seems counterintuitive. This behavior is likely due to the presence of smaller objects and edges (e.g. during systole), which are represented by high frequency *k-*space content. Further accentuating this point is the reduced MSE for our two-stage Kalman filter simulations with Cartesian sampling. This is expected, as Cartesian sampling emphasizes equally all frequency regions of *k*-space. However, we must remember that despite the quantitative value of the MSE metric, the human visual system emphasizes low spatial frequency content and judges image quality in a manner difficult to capture faithfully using mathematical constructs.

A protocol for building the predictive signal model is crucial for the success of our two-stage Kalman filter, especially when reconstructing at a higher temporal resolution, and it must be designed judiciously. This makes logical sense, as the performance of any Kalman filter is tied to the accuracy of $f({x}_{t-1})$ and $Q$. The training set used to estimate $f({x}_{t-1}^{a})$ must be an accurate descriptor for the underlying cardiac dynamics. This is a natural limitation of statistical estimation and is the primary motivation for performing the training scan immediately prior to the test scan, as demonstrated via previous work. Unfortunately, due to usage of the UK Biobank data, this was not possible in our studies. It should be noted that any inaccuracy within the training set would yield inaccuracies in our estimation of $f({x}_{t-1}^{a})$, which could cause divergence^8^. Spikes in the MSE graphs serve as a reminder of this fact. Again, these spikes are likely due to an increased presence of high frequency k-space content, which is difficult to estimate compared to low frequency k-space content. This is illustrated within the error images.

In regards to the two-stage CS scheme, the signal model proved essential in removing the streaking artifacts caused by inadequate sampling of k-space. While an undersampling factor of 8.83 may not appear large at first glance, one must remember that only a single coil was used during the reconstructions, forgoing any additional acceleration and quality that may be gained from multicoil imaging. Coupled with the usage of a Cartesian undersampling mask, streaking artifacts are to be expected. The successful removal of these artifacts depends on an accurate state-space consistency term, as well as a well-tuned weighting factor ${\lambda }_{ss}$. Care must also be taken to avoid overfitting to the predictive signal model as well, which is controlled by the tuning of ${\lambda }_{ss}$.

Various factors could lead to an inaccurate predictive signal model, including: inherent variations between different MRI experiments, inaccurate phase estimation, motion artifacts, and noise in the training scan. To help illustrate the consequences of an inaccurate predictive signal model, consider the random-walk Kalman filter implementation for the secondary scenarios. The appearance of perturbations is due to inaccurate modeling; the training set for this scenario consisted of $T=10$ images, whereas one cardiac cycle of the test set consisted of $T=50$ images. Inaccurate modeling results in a reliance on the mean value, hence, the perturbations. This may also explain the random-walk Kalman filter reconstruction artifacts in Figs. 2, 3, 4 and 5. Thus, diligence in all aspects of building the predictive signal model is essential.

Fortunately, strategies exist to deal with each of the above examples. To combat variations between MRI experiments, one should acquire the training set during the same experiment as the test set. This is certainly feasible if one chooses to use our algorithms, as the training set need not be large. A second solution to combat variations between MRI experiments is to use the DC value of k-space to equate the training and test set means, as implemented in our work. Regarding motion artifacts, multiple strategies have been developed^24,27–29^. Implementing motion compensation would be essential, as motion could introduce a spatial offset not accounted for in the training set. Lastly, our simulations demonstrate the benefits of denoising images a priori via a convolutional neural network.

An important limitation regarding our predictive signal model is the usage of CINE data. Reorganizing cardiac CINE frames is not a perfect model for arrhythmic events, especially if said event consists of both temporal and physiological changes (e.g. atrial fibrillation). This was the motivation for developing an algorithm capable of handling *any* arbitrary change in the temporal resolution of the test scan. Demonstrating the ability to handle a non-periodic sinus rhythm is important, as conventional CINE precludes data should the heart rate change mid-acquisition. Furthermore, our protocol for estimating $Q$ and tuning ${\lambda }_{ss}$ was designed to accommodate variability, such as physiological changes during arrhythmic events. Our second secondary simulation was also designed to examine the behavior of $Q,$ and did provide confirmation that a poorly chosen $Q$ will result in divergence of our two-stage Kalman filter.

Let us revisit the strategies for acceleration: undersampling, accommodating for low temporal resolution training data, and computational time. Theoretically, the undersampling rate can be increased at the cost of an increased MSE. This tradeoff may be acceptable if the MSE of our algorithms remain well below that of their conventional counterparts. Based on the Kalman filtering results, an undersampling rate of 12.5 appears to strike a good balance on average and is higher than that commonly found in the current literature^6^. The second strategy, accommodating for low temporal resolution training data, can be adjusted to compensate for any arbitrary difference in temporal resolution between the training and test set. The generalizability of our algorithm in this manner is a potential asset for dynamic CINE MRI. Computational times are another consideration for further optimization, as clinical use demands that reconstruction times of the Kalman filter be further reduced. Although we did not optimize our Kalman filter implementation, we can turn to multiple strategies in the literature^30–32^ to optimize computationally intensive steps, such as computing a Moore–Penrose pseudoinverse.

Let us further explore the reduction of computational time. As established in the introduction, multiple approaches for dynamic real-time cardiac MRI exist, many of which exhibit clinically feasible computational times. Our algorithm’s novelty is computationally demanding, as significant resources are required to accurately model irregular cardiac dynamics for an arbitrary temporal resolution and sinus rhythm. This was particularly noticeable in the OCMR reconstructions, as those images were significantly larger than the images used for the UK Biobank simulations. However, our algorithm’s ability to accommodate unforeseen cardiac dynamics *without* the need for rebinning or preclusion of *k*-space is an asset for dynamic cardiac MRI. In the case of Kalman filtering, this is further augmented by the Kalman filter’s innate ability to ameliorate noise introduced due to high acquisition speeds. An immediate next step would be to mathematically simplify the components of our modelling process and our Kalman filters. For example, previous work assumed multiple matrices were diagonal, thereby allowing them to reduce the computational time required. While diagonalization is not a valid approach here (as the off-diagonal entries are important), it does illustrate that mathematical simplification is certainly feasible.

One of the immediate next steps is to implement our technique on an MRI scanner for feasibility testing and validation. This would address multiple limitations: lack of access to raw *k*-space data in different formats, inability to perform the training and test scan in the same session, imperfect modelling of arrhythmic events, and inability to fully investigate the importance of selecting $Q$. Furthermore, implementation on an MRI scanner is necessary for proper validation of clinical measures such as left ventricular ejection fraction. In vivo, we can robustly build the predictive signal model by obtaining a single fully sampled CINE cardiac cycle prior to real-time data acquisition.

Another interesting future study would be to improve the training scan. While the requirement of a single cardiac cycle is advantageous for training, usage of fully sampled *k-*space does pose some questions regarding our algorithm’s flexible undersampling. Furthermore, it is important to recognize that all training scans will likely be subjected to motion artefacts or inconsistencies. Implementing a solution that does not utilize fully sampled *k-*space while ensuring artefacts are compensated for is an immediate next step and would pose an excellent future study in tandem with the study described in the previous paragraph.

## Conclusion

We have presented a novel predictive signal model for dynamic CMR that can be used to obtain high-quality reconstructions at an average acceleration of 12.5 from undersampling and another factor of 5 from temporal interpolation. We demonstrate the ability to use this algorithm in multiple reconstruction frameworks, an asset for dynamic CMR. With its attributes of adaptability to irregular heart rhythms, versatile undersampling, ability to accommodate for low temporal resolution training data, and the ability to be integrated with previously developed techniques, this predictive signal model algorithm can serve as a foundation for further innovations in robust, real-time dynamic CMR.

## Supplementary Information

Supplementary Figure S1.

Supplementary Figure S2.

Supplementary Video S1.

Supplementary Video S2.

## Data Availability

For all UKBiobank datasets, please visit https://www.ukbiobank.ac.uk/; interested parties must obtain permission from UKBiobank in order to download these datasets. We cannot provide a direct link to these datasets, as doing so would violate the strict confidentiality specified in the research agreement between our laboratory and the UKBiobank. For all Ohio State University datasets, please visit https://ocmr.info/.

## References

[1] Ghugre NR, Pop M, Barry J, Connelly KA, Wright GA (2013). Quantitative magnetic resonance imaging can distinguish remodeling mechanisms after acute myocardial infarction based on the severity of ischemic insult. Magn. Reson. Med..

[2] Tseng W-YI, Su M-YM, Tseng Y-HE (2016). Introduction to cardiovascular magnetic resonance: Technical principles and clinical applications. Acta Cardiol. Sin..

[3] Uecker M (2010). Real-time MRI at a resolution of 20 ms. NMR Biomed..

[4] Kellman P (2009). High spatial and temporal resolution cardiac cine MRI from retrospective reconstruction of data acquired in real time using motion correction and resorting. Magn. Reson. Med..

[5] Xue H, Kellman P, Larocca G, Arai AE, Hansen MS (2013). High spatial and temporal resolution retrospective cine cardiovascular magnetic resonance from shortened free breathing real-time acquisitions. J. Cardiovasc. Magn. Reson..

[6] Curtis AD, Cheng HM (2022). Primer and historical review on rapid cardiac CINE MRI. J. Magn. Reson. Imaging.

[7] Kay SM (2013). Fundamentals of Statistical Signal Processing (III): Practical Algorithm Development.

[8] Jwo DJ, Cho TS (2007). A practical note on evaluating Kalman filter performance optimality and degradation. Appl. Math. Comput..

[9] Sümbül U, Santos JM, Pauly JM (2009). Improved time series reconstruction for dynamic magnetic resonance imaging. IEEE Trans. Med. Imaging..

[10] Vaswani, N. *KF-CS: Compressive Sensing on Kalman Filtered Residual* (2009).

[11] Feng X, Salerno M, Kramer CM, Meyer CH (2013). Kalman filter techniques for accelerated Cartesian dynamic cardiac imaging. Magn. Reson. Med..

[12] Forman, C., Wetzl, J., Hayes, C. & Schmidt, M. Compressed sensing: A paradigm shift in MRI. *MAGNETOM Flash* (2016).

[13] Liu, J. *et al.* Dynamic cardiac MRI reconstruction with weighted redundant Haar wavelets. In *Proc. 20th Annual Meeting of ISMRM, Melbourne, Australia* (2012).

[14] Geerts-Ossevoort, L. *et al.* Compressed SENSE speed done right. Every time. *Philips FieldStrength Magazine* (2018).

[15] Donoho DL (2006). Compressed sensing. IEEE Trans. Inf. Theory.

[16] Candès EJ, Romberg J, Tao T (2006). Robust uncertainty principles: Exact signal reconstruction from highly incomplete frequency information. IEEE Trans. Inf. Theory.

[17] Antun V, Renna F, Poon C, Adcock B, Hansen AC (2020). On instabilities of deep learning in image reconstruction and the potential costs of AI. Proc. Natl. Acad. Sci. U.S.A..

[18] Feng L (2014). Golden-angle radial sparse parallel MRI: Combination of compressed sensing, parallel imaging, and golden-angle radial sampling for fast and flexible dynamic volumetric MRI. Magn. Reson. Med..

[19] Ward BD, Janik J, Mazaheri Y, Ma Y, DeYoe EA (2012). Adaptive Kalman filtering for real-time mapping of the visual field. Neuroimage..

[20] Zhao L, Feng X, Meyer CH (2016). Direct and accelerated parameter mapping using the unscented Kalman filter. Magn. Reson. Med..

[21] Terejanu, G. *Extended Kalman Filter Tutorial*. *Technical Report: Extended Kalman Filter Tutorial* (2003).

[22] Sengupta SK, Kay SM (1995). Fundamentals of statistical signal processing: Estimation theory. Technometrics.

[23] Tsao J, Boesiger P, Pruessmann KP (2003). k-t BLAST and k-t SENSE: Dynamic MRI with high frame rate exploiting spatiotemporal correlations. Magn. Reson. Med..

[24] Zhou R (2019). Free-breathing cine imaging with motion-corrected reconstruction at 3T using SPiral acquisition with respiratory correction and cardiac self-gating (SPARCS). Magn. Reson. Med..

[25] Chen, C. *et al.**OCMR (v1.0)—Open-Access Multi-coil k-Space Dataset for Cardiovascular Magnetic Resonance Imaging* (2020).

[26] Block Aus Mainz, K. T. *Advanced Methods for Radial Data Sampling in Magnetic Resonance Imaging* (2008).

[27] Stadler A, Schima W, Ba-Ssalamah A, Kettenbach J, Eisenhuber E (2007). Artifacts in body MR imaging: Their appearance and how to eliminate them. Eur. Radiol..

[28] Ludwig J, Speier P, Seifert F, Schaeffter T, Kolbitsch C (2021). Pilot tone-based motion correction for prospective respiratory compensated cardiac cine MRI. Magn. Reson. Med..

[29] Ghodrati V (2021). Retrospective respiratory motion correction in cardiac cine MRI reconstruction using adversarial autoencoder and unsupervised learning. NMR Biomed..

[30] Raitoharju M, Piche R (2019). On computational complexity reduction methods for Kalman filter extensions. IEEE Aerosp. Electron. Syst. Mag..

[31] Pourasad Y, Vahidpour V, Rastegarnia A, Ghorbanzadeh P, Sanei S (2022). State estimation in linear dynamical systems by partial update Kalman filtering. Circuits Syst. Signal Process..

[32] Kaniewski P (2020). Extended Kalman filter with reduced computational demands for systems with non-linear measurement models. Sensors.

