# Supplementary figures and images for: A predictive signal model for dynamic cardiac magnetic resonance imaging
Source: Sci Rep. 2023 Jun 25;13:10296. doi: 10.1038/s41598-023-37475-5 (PMC10290992; doi:10.1038/s41598-023-37475-5)

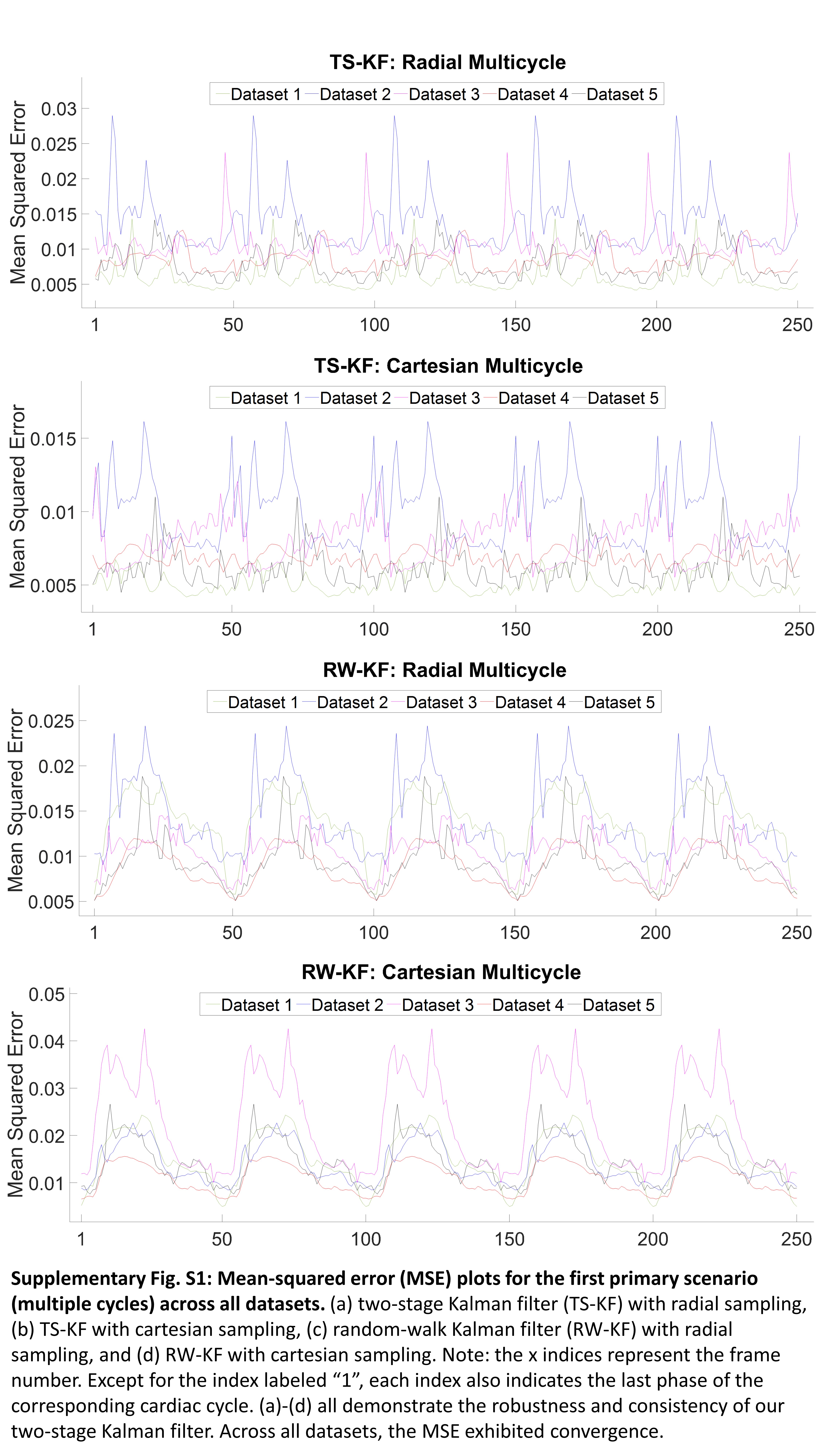

Supplement: Supplementary file 1 — Supplementary Figure S1. [file 41598_2023_37475_MOESM1_ESM.jpg]

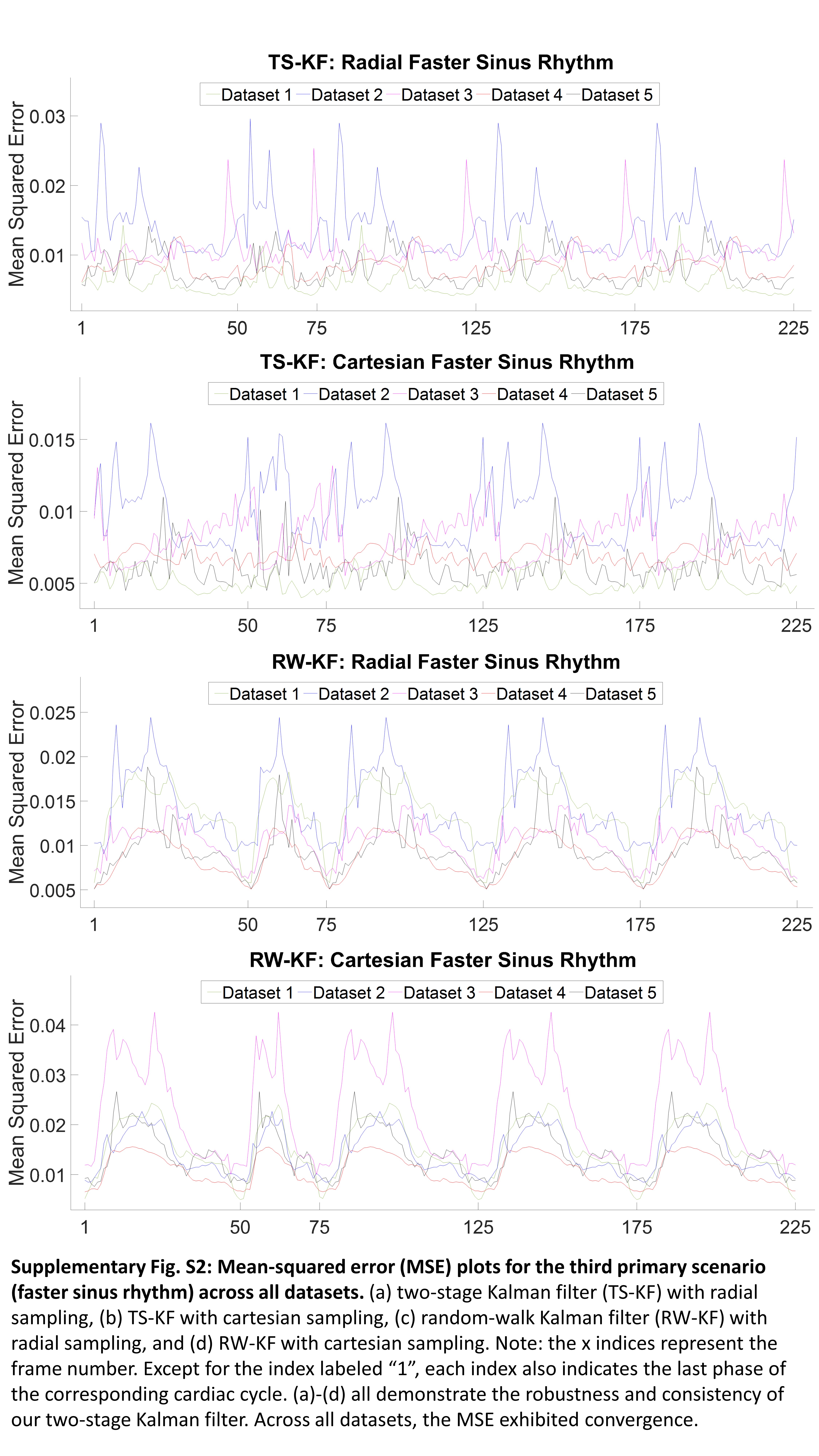

Supplement: Supplementary file 2 — Supplementary Figure S2. [file 41598_2023_37475_MOESM2_ESM.jpg]
